# Combining Global Positioning System (GPS) with saliva collection among sexual minority adults: A feasibility study

**DOI:** 10.1371/journal.pone.0250333

**Published:** 2021-05-06

**Authors:** Tzuan A. Chen, Nathan Grant Smith, Seann D. Regan, Ezemenari M. Obasi, Kathryn Freeman Anderson, Lorraine R. Reitzel

**Affiliations:** 1 HEALTH Research Institute, University of Houston, Houston, Texas, United States of America; 2 Department of Psychological, Health, and Learning Sciences, University of Houston, Houston, Texas, United States of America; 3 Department of Epidemiology, Columbia University Mailman School of Public Health, New York, New York, United States of America; 4 Department of Sociology, University of Houston, Houston, Texas, United States of America; SUNY Downstate: SUNY Downstate Health Sciences University, UNITED STATES

## Abstract

**Background:**

This is the first study, of which we are aware, to evaluate the feasibility and accessibility of simultaneous use of Global Positioning System (GPS) and saliva collection for biomarker assessment as an objective measure of stress physiology among sexual minority (lesbian, gay, bisexual, queer, and other non-heterosexual identities) individuals. The principal motivation for pairing GPS and saliva collection was to investigate how characteristics of the built and social environments along with participants’ daily activity paths affect stress. This can contribute to a better understanding of health and health behaviors in the sexual minority community.

**Methods:**

A convenience sample of enrolled participants (N = 124) from Houston, Texas was asked to complete questionnaires, carry with them a GPS unit daily, and collect and store 6 samples of saliva at specific times across the span of a day prior to a second visit around one week later.

**Results:**

Of 124 participants, 16 participants (12.90%) provided no useable GPS data and 98 (79.03%) provided at least 4 days of data. More than three-fourths (n = 98, 79.03%) also provided complete saliva samples.

**Conclusions:**

Our results show that the simultaneous use of GPS and saliva collection to assess sexual minority individuals’ activity paths and stress level is feasible.

## Introduction

Spatial epidemiology is a fast-growing field, a modern adaptation of the established methodology of geographic analysis, which dates to the 18^th^ century [1]. Traditionally, epidemiologic research focuses on person and time, with little attention to the implications of place and space due to project design, data limitations, etc. [2]. However, the built (e.g., proximity and density of alcohol and tobacco retail outlets in a neighborhood) and social (e.g., crime rates, racial composition of a neighborhood, etc.) environments are critical factors affecting health behaviors and health outcomes [3, 4]. Many studies have shown the importance of activity space and residential context on the incidence and prevalence of risk behaviors among populations vulnerable to health disparities in the United States [5–12], including among sexual minorities (those identifying as lesbian, gay, bisexual, queer, or other non-heterosexual identities) [13, 14].

Over recent decades, the application of technology has enriched the complexity, sophistication, and utility of spatial epidemiology [15–17]. The development of geographic information systems (GIS) has provided a powerful capability for researchers to rapidly examine spatial patterns and processes. GIS data and spatial methods have been used in many epidemiologic applications such as disease mapping, rate smoothing, and spatial modeling [18]. With the rapid increase in computing power, advancement in technologies have extended GIS capacities. For example, the availability of low-cost personal Global Positioning Systems (GPS) has made their use increasingly popular—a development that has been discussed in many recent health research studies [9, 19, 20]. GPS tracking generates geographic data, which allows for near continuous monitoring of mobility (i.e., activity paths) across space. GPS technology not only allows researchers to investigate the impact of spatial contexts when wedded with GIS data, but also to consider the timing and the duration of exposure within spatial contexts [21, 22].

GPS technology has been widely used among diverse populations [7, 9, 14, 23–25] to assess contextual determinants and correlates of different health behaviors such as health-related product purchasing behaviors, physical activity, dietary intake, and smoking behaviors [10, 12, 23, 26]. However, some studies indicate that participants have concerns regarding confidentiality and privacy when GPS data are collected that may affect the acceptability of these procedures [27, 28]. This may especially be the case for population subgroups that have been historically marginalized or mistreated. This indicates the need to assess the feasibility and acceptability of wearing GPS devices among sexual minority individuals. Additionally, there is a need to examine whether or not GPS technology can be readily paired with data on stress and negative emotion. Pairing GPS and affective data has the benefits of better understanding how the external context may affect internal experiences.

Historically, data measuring experiences of mood and stress have been collected via paper and pencil questionnaires that rely on the retrospective recall of thoughts and feelings experienced over a specified timeframe (e.g., previous week, month, year, etc.). Recognizing the limitations of memory recall and the potential for response bias, there has been a push for objective methodological innovations in naturalistic studies where participants navigate their environment while collecting biomarker samples (e.g., saliva)–an objective neuroendocrine assessment–that can be paired with the real time measurement of mood at several timepoints across the day [29]. Leveraging Ecological Momentary Assessment (EMA) methodology to collect mood data on a smartphone–or equivalent device–can better capture real time fluctuations in self-reported mood that can be paired with neuroendocrine reactivity (e.g., cortisol) while minimizing measurement error [30].

Many studies have shown that salivary cortisol represents a nonintrusive assessment of physiological stress reactivity that has been cross validated with serum cortisol in naturalistic settings, which allows for the simultaneous measurement of diurnal cortisol and the associated time-varying fluctuation in physiologic stress responses across a specified timeframe [31–44]. More specifically, higher levels of salivary cortisol were associated with increased levels of self-reported stress and negative emotions [31, 32]. Similar results were observed by other studies conducted with sexual minority participants [37–40]. Prior work suggested there were no differences in diurnal cortisol rhythms across sexual orientation groups [37], possibly due to the exhibition of physiologic resilience of the sexual minority participants, whereas differences were observed by race/ethnicity [38]. While there is a dearth in the amount of neuroendocrine studies that have focused on this population subgroup [37–44], a recent study (Growing Up Today Study, GUTS) by Austin and colleagues found a low response rate of young lesbian, gay and bisexual individuals providing saliva samples [37]. Thus, the literature would benefit from additional feasibility studies examining saliva sample collection among sexual minorities in naturalistic environments, particularly when such protocols include other procedures that may impact a person’s choice to participate.

Attitudes toward sexual minorities have changed remarkably in the United States during the past three decades; for example, the National Institute of Health now recognizes sexual minorities as a health disparities group and there is greater appreciation for understanding their collective experience that oftentimes diverge from heterosexual norms [45, 46]. For example, sexual minorities experience higher risks for many negative physical and mental health outcomes (e.g., chronic disease, human immunodeficiency virus (HIV) infection, and anxiety disorders [47–51]), and non-health promoting behaviors (e.g., smoking, heavy drinking, and illicit drug use [50]) relative to heterosexuals, with some variation between sexual minority sub-groups. Chronic exposure to stress represents a transdiagnostic vulnerability that is linked to health disparities experienced by marginalized communities [50–56]. Increased stress levels may be triggered by many factors including incessant experiences of discrimination and homophobia. Growing evidence suggests sexual minorities are exposed to more stress than their heterosexual counterparts [52–55]. The health disparities experienced among sexual minorities has been conceptualized through the minority stress theory that suggests that discrimination, stigma, and prejudice create a stressful social environment, which can in turn cause mental or physical health issues [56]. Specifically, Meyer’s minority stress model posits that sexual minorities are exposed to myriad and detrimental stressors related to social stigma and discrimination, which in turn contribute to the higher prevalence of health-risk behaviors and associated disorders and diseases [57–60]. A meta-analysis [56] found lesbian, gay, and bisexual individuals were more likely to have a mood, anxiety, or substance abuse disorder in their lifetime when compared to heterosexual individuals.

There are published studies that have collected salivary biomarkers among sexual minorities in a naturalistic setting [37–40, 61]; however, we were unaware of studies that have specifically combined the use of GPS and saliva collection among this group. To address this gap in the literature, we assess the feasibility of coupling GPS technology with objective salivary biomarker collection in this population. Tracking activity paths using GPS is a novel approach that provides researchers with an objective assessment of physical location where people interact with the built and social environmental contexts without the need for retrospective recall. GPS technology has the capacity to assess spatial mobility and polygamy in real time to understand the characteristics of participant activity spaces across contexts of interest (e.g., built or social environmental exposures) through the automated collection of latitude and longitude data [62–66]. Likewise, assessing stress physiology using inexpensive saliva collection protocols provides an objective measure of neuroendocrine reactivity to stress that is easy to collect and less invasive than to other modalities (i.e., serum via venipuncture). Moreover, stress-related steroid hormones like cortisol could then be paired with real time objective (e.g., GPS) and self-report data (e.g., perceived stress, mood, etc.) to provide a more comprehensive insight into the experience of sexual minority groups grappling with chronic exposure to stress in their day-to-day lives.

It is imperative that sexual minorities–a health disparity population–be included in cutting-edge research that can identify novel mechanisms for understanding how activity space couples with mood to have a deleterious effect on stress dysregulation–a known risk factor for chronic diseases. Integrating inexpensive GPS technology with a salivary biomarker collection protocol provides an innovative opportunity for quantifying stress physiology in real time as sexual minorities navigate the real-world environment. Despite increasing acknowledgements of the importance of the built and social environments for health, the feasibility of pairing the use of a GPS device with salivary biomarker collection in these communities remains poorly understood.

The purpose of this study was to assess the feasibility of recruiting adults identifying as sexual minorities to participate in a study involving: (1) location and wear-time data using a GPS monitor in their natural setting; (2) collection of saliva-based biomarker samples at specified times throughout a single day; and (3) assessment of participant characteristics that may inform within-group variation in the acceptability and feasibility of such methodological protocols.

## Materials and methods

### Eligibility and recruitment

Project FRESH AIR (Focused Research to Enhance Social Health Among Individuals in the Rainbow) was designed to better understand how psychosocial stressors affect self-reported health and health behaviors among adults identifying as sexual minorities. This project was unique in its combination of several data sources including self-report questionnaires, anthropometric measurement, carbon monoxide assessment, GPS tracking, and salivary cortisol assaying among sexual minority individuals. Eligibility criteria included aged 18 years or older, self-reported sexual minority, provision of valid contact information, and self-reported willingness to comply with the study protocol. Those who were pregnant, lactating, possibly pregnant, or taking necessary prescription medication that could not be postponed during the execution of the saliva collection protocol (i.e., delayed until after the last saliva sample of the day was collected) were excluded from participation due to the potential effects on assay results.

Recruitment was achieved by the distribution of study flyers at accommodating businesses and healthcare centers within neighborhoods with a high proportion of sexual minority adults (e.g., Montrose, a historically sexual minority enclave of Houston). We also advertised on a university campus, within sexual minority-targeted magazines (e.g., Outsmart), online (e.g., Scruff), and on internal (university) television screens. The goal was to recruit 124 participants based on initial power estimates and funding for study procedures. Of those who initiated contact with the study team to be screened (N = 200), 18 individuals could not be reached back by phone. Of those screened, 164 individuals met eligibility criteria. Screened individuals were ineligible primarily due to medication (39%), lack of interest (22%), and heterosexuality (17%). Of the 164 individuals screened eligible, 40 did not show for their visit 1. No shows were called and/or emailed, on average, 4 times in attempts to reschedule. Participants were primarily recruited through information from friends or other participants (n = 53, 42.74%), followed by sexual minority-focused healthcare and social centers (n = 30, 24.19%), other/not provided (n = 26, 20.97%), and print and electronic advertisement (n = 15, 12.10%). Study procedures were approved by the IRB at the University of Houston and written informed consent was obtained from all participants.

### GPS protocol and data processing procedures

Participants were required to make two visits to the University of Houston. During visit 1, they were screened, consented, and enrolled. They were assigned a GPS monitoring device to carry between study visits. During visit 2, approximately one week later, participants returned the GPS unit. The GPS device was used to monitor participants’ location to gain a better understanding of the potential influence of environmental context on participants’ health and health behaviors. The GPS provided data on where participants spent most of their time, allowing future analysis to examine exposure to environmental factors in those places (e.g., tobacco outlet density and proximity, fast food density and proximity, as well as broader food environment, alcohol/bar exposure, etc.). As this protocol was designed to measure feasibility, there was no minimum number of days required for GPS collection; rather, days of collection were dependent on the participants’ availability to return for the 2^nd^ visit. Participants were compensated with Target gift cards with a value of $30 at visit 1 for the completion of study procedures and a value of $20 at visit 2 for returning the GPS device. Researchers used the i-gotU GT-120 travel and sports GPS logger device (MobileAction, Taipei, Taiwan), which has been used in previous research [67–69]. Participants were asked to carry the small GPS device during all times except when bathing, swimming, and sleeping. They were provided with a lanyard to wear it around their neck. Participants were also asked to charge the device nightly and were provided with a charging cord. In addition to verbal explanation, participants were provided with a “take home” card reiterating these instructions.

All GPS devices were associated with a unique ID number that was different from the participant ID number. Due to battery drain considerations, the tracking interval of the GPS devices used in this study was set at 15 minutes. At visit 2, project staff downloaded the GPS data on a single password-protected computer in a locked research lab. The GPS devices used in this study were by design “passive,” meaning the location data of participants could not be seen in real time. The research team uploaded GPS data to encrypted project computers and used GIS software (ESRI ArcGIS 10.6) for post-processing.

For the purposes of this feasibility study, we defined complete GPS data as having 4 or more days of data. Having 4 or more days of GPS data provides some variability whereby consistency of activity paths may be determined, and, in many cases, both weekdays and weekend data could be captured.

### Saliva cortisol collection procedures

While evidence suggests that reduced sampling protocols (e.g., studies collecting 4 or fewer samples per day) were sufficient to measure diurnal cortisol slopes and cortisol awakening response, evidence also indicates that more time points are necessary for adequate estimates of the area under the curve [70, 71], which is an important indicator for psychological and physiological functioning [29]. Therefore, between visits 1 and 2, participants were asked to provide 6 saliva samples by passive drool through a straw into a previously labeled cryovial tube [72, 73]–at specified times across a single day: (1) when waking up and before getting out of bed; (2) 30 minutes after waking up before brushing teeth; (3) 90 minutes after waking up; (4) at approximately 2:00 p.m. or an hour after lunch; (5) at approximately 5:00 p.m. or before dinner; and (6) before going to bed. Samples 1–3 were aimed to model the cortisol awaking response. Samples 4 and 5 were collected as indicators of the diurnal down-regulation of the hypothalamic–pituitary–adrenal (HPA) axis through the early evening. Lastly, the final sample was collected to identify resting cortisol representing a potential lower-limit since hormone levels are typically down regulated to a low-point prior to going to sleep [74, 75].

Saliva samples were collected to study the fluctuation of stress-related biomarkers throughout the day. Each participant was given a saliva kit, collection diary, and detailed instructions for collection and storage during visit 1. Participants collected the sample by using the provided straw to passively drool into a cryovial that was labeled with their participant ID and sample number. Moreover, participants were instructed to rinse their mouths with water and wait 5 minutes if their mouth was too dry to provide saliva sample. Participants were instructed not to eat, drink, or smoke cigarettes 30 minutes before collecting a sample. Participants were instructed to store their saliva samples in their freezer until visit 2. Participants received a small insulated cooler and an ice pack that would facilitate storage and transport of the samples to the University of Houston under temperature-controlled conditions. In addition to verbal explanation, participants were provided with a "take home" card reiterating these instructions.

Participants received a “Collection Diary” in which they were asked several questions about each saliva collection, including time taken, duration of procedure, mood, and if anything unusual or stressful had occurred that day. Participants returned the Collection Diary, cooler, ice pack, and saliva samples at visit 2. During this time, research personnel asked a few questions about the saliva collection process (e.g., were they able to freeze the sample immediately? were there any problems with collection?), and noted answers that might compromise the integrity of the samples. Participants were compensated $5 in Target gift cards for each saliva sample returned at visit 2. All saliva samples were immediately stored in a -30°C laboratory freezer until being assayed. Research personnel entered Collection Diary data into a spreadsheet by hand and determinations were made about integrity of samples based on information provided by the participant and visual inspection of the vials. Reported time to collect the saliva samples ranged from 1–22 minutes as reported by participants in collection diary. Average time to collect the samples by collection vial ranged from 2.38 (±1.94) minutes to 3.09 minutes (±2.96), with earlier samples generally taking a longer average time until completion than later samples. Additionally, almost all the self-reported collection diaries received suggested no violations of the instruction of no drinking or tobacco use 30 minutes prior to sample collections, with only one participant clearly reported drinking alcohol 30 minutes prior to one sample collection. This, represented, overall, 0.17% of completed saliva samples provided to the study team. Very few participants ate food within 30 minutes prior to sample collection with 4 (3.23%), 2 (1.61%), 3 (2.42%), and 1 (0.81%) participants violated this rule in sample 2, 3, 5, and 6 collection, respectively. This, represented, overall, 1.7% of completed saliva samples provide to the study team. Only small percent of participants, ranging from 1 (0.81%, Sample 6) to 6 (4.84%, Sample 1) participants, reported they were not able to put the sample in the freezer right away. This represented, overall, 2.89% of completed saliva samples provided to the study team.

### Socio-demographic characteristics

Information concerning participants’ sociodemographic characteristics was collected, including age, gender (cisgender male, cisgender female, or transgender), sexual orientation (monosexual vs. bisexual), race (Asian, Black/African American, Latino, Non-Latino White, and Other/Multiracial), and education (≤ high school education vs. trade school/college degree). Gender, sexual orientation, race, and education were recoded into binary variables for ease of reporting.

### Statistical analysis

The descriptive statistics regarding the participants’ characteristics and recruitment information were generated. To assess the feasibility and acceptability of GPS device and saliva collection, two binary variables were generated indicating the completion status of GPS device (< or ≥ 4 days) and saliva collection (collected all 6 saliva samples or not). Study participants were classified into 1 of 4 GPS/saliva compliance groupings based on their data completion: 1) GPS alone (≥ 4 days), 2) saliva data alone (collected all 6 saliva samples), 3) both GPS and saliva data (i.e., ≥ 4 days of GPS data and collected all 6 saliva samples), and 4) neither GPS nor saliva data. Analysis of variance and Fisher’s exact test were used to examine the differences by these groups in participants’ age and other sociodemographic characteristics, respectively. Analyses were conducted separately for those with complete saliva data (i.e., 6 samples returned) based on whether or not they reported potential violations of instructions for 1 or more of the samples in their Collection Diary (e.g., ate food within 30 min prior to sample collection). Additionally, socio-demographic differences between those providing complete 6 saliva samples (i.e., complete saliva data alone group and complete GPS and saliva data group) with no potential violations vs. those with a potential violation of at least 1 sample were examined. For those participants missing saliva samples, it could be informative to know which samples were missing (e.g., monotone or non-monotone), and the incidence of missing samples. Therefore, descriptive statistics and missingness of saliva collection were provided. In addition, GPS data, including the number of days of data and valid GPS points/day per participant, were presented. All statistical analyses were conducted using SAS 9.4 [76].

## Results

### Overall

Of the 124 participants, 70.97% (n = 88) participants had complete GPS and saliva data; 8.06% (n = 10) participants had complete GPS data but not saliva data; 8.06% (n = 10) participants had complete saliva data but not GPS data; and 12.90% (n = 16) participants did not have either GPS or saliva data. Of the 124 participants, the average age was 36.59 (±14.86) years and 50% were cisgender male; 52.42% were monosexual; 39.84% reported being Black/African American; 36.59% reported being non-Latino White; and 65.32% reported a high school education or less education (Table 1). No significant differences were found in participants’ characteristics across the aforementioned 4 GPS/saliva compliance groupings.

**Table 1:** Characteristics of participants by different types of data completion on participants providing entire 6 saliva samples including those who violated directions.

	Total (n = 124)	Complete GPS Alone (n = 10)	Complete Saliva Alone (n = 10)	Complete GPS and Saliva (n = 88)	Neither GPS nor Saliva (n = 16)	*p-*value^a^
	M (SD)/% [n]	M (SD)/% [n]	M (SD)/% [n]	M (SD)/% [n]	M (SD)/% [n]	
Age	36.59 (14.86)	39.80 (8.79)	33.10 (10.49)	36.05 (15.95)	39.75 (14.02)	0.6072
Gender						0.1826
Cisgender Male	50.00 [61]	50.00 [5]	20.00 [2]	50.57 [44]	66.67 [10]	
Cisgender Female	31.97 [39]	30.00 [3]	30.00 [3]	33.33 [29]	26.67 [4]	
Transgender	18.03 [22]	20.00 [2]	50.00 [5]	16.09 [14]	6.67 [1]	
Sexual Orientation						0.6189
Monosexual	52.42 [65]	50.00 [5]	60.00 [6]	54.55 [48]	37.50 [6]	
Bisexual	47.58 [59]	50.00 [5]	40.00 [4]	45.45 [40]	62.50 [10]	
Race/Ethnicity						0.6703
Asian	3.25 [4]	0.00 [0]	10.00 [1]	3.41 [3]	0.00 [0]	
Black/African American	39.84 [49]	60.00 [6]	20.00 [2]	37.50 [33]	53.33 [8]	
Latino	15.45 [19]	20.00 [2]	10.00 [1]	17.05 [15]	6.67 [1]	
Non-Latino White	36.59 [45]	20.00 [2]	50.00 [5]	36.36 [32]	40.00 [6]	
Other/Multiracial	4.88 [6]	0.00 [0]	10.00 [1]	5.68 [5]	0.00 [0]	
Education						0.5289
≤ High School Diploma	65.32 [81]	60.00 [6]	60.00 [6]	63.64 [56]	81.25 [13]	
Trade School/College Degree	34.68 [43]	40.00 [4]	40.00 [4]	36.36 [32]	18.75 [3]	

Note. This table includes participants providing entire 6 saliva samples including those who violated directions.

^a^Analysis of variance and Fisher’s Exact test were used to compare between 4 groups in age and all the other characteristics, respectively.

More than three-fourths of the participants (n = 98, 79.03%) provided all 6 saliva samples. There were 4 (3.23%), 2 (1.61%) and 1 (0.81%) participants who provided only the initial one, the initial two, or three saliva samples, respectively. There were 19 participants (15.32%) who did not provide any saliva samples. Overall, 24 participants potentially violated the directions for saliva collection and were further excluded. Of the 100 participants who did not violate directions, there were no significant differences in socio-demographic variables across the 4 GPS/saliva compliance groups (Table 2). Additionally, there were no socio-demographic differences between violators (24 = 124–100) and non-violators (74 = 5+69) with the exception of age. The violators were significantly younger than non-violators (28.25 vs. 38.18, p = 0.0057) (Table 3).

**Table 2:** Characteristics of participants by different types of data completion on participants providing entire 6 saliva samples without directions violated.

	Total (n = 100)	Complete GPS Alone (n = 10)	Complete Saliva Alone (n = 5)	Complete GPS and Saliva (n = 69)	Neither GPS nor Saliva (n = 16)	*p-*value^a^
	M (SD)/% [n]	M (SD)/% [n]	M (SD)/% [n]	M (SD)/% [n]	M (SD)/% [n]	
Age	38.59 (15.2)	39.80 (8.79)	38.20 (11.14)	38.17 (16.58)	39.75 (14.02)	0.9766
Gender						0.2275
Cisgender Male	51.02 [50]	50.00 [5]	20.00 [1]	50.00 [34]	66.67 [10]	
Cisgender Female	33.67 [33]	30.00 [3]	20.00 [1]	36.76 [25]	26.67 [4]	
Transgender	15.31 [15]	20.00 [2]	60.00 [3]	13.24 [9]	6.67 [1]	
Sexual Orientation						0.7533
Monosexual	49.00 [49]	50.00 [5]	60.00 [3]	50.72 [35]	37.50 [6]	
Bisexual	51.00 [51]	50.00 [5]	40.00 [2]	49.28 [34]	62.50 [10]	
Race/Ethnicity						0.5694
Asian	4.04 [4]	0.00 [0]	20.00 [1]	4.35 [3]	0.00 [0]	
Black/African American	43.43 [43]	60.00 [6]	20.00 [1]	40.58 [28]	53.33 [8]	
Latino	14.14 [14]	20.00 [2]	0.00 [0]	15.94 [11]	6.67 [1]	
Non-Latino White	33.33 [33]	20.00 [2]	40.00 [2]	33.33 [23]	40.00 [6]	
Other/Multiracial	5.05 [5]	0.00 [0]	20.00 [1]	5.80 [4]	0.00 [0]	
Education						0.5145
≤ High School Diploma	65.00 [65]	60.00 [6]	60.00 [3]	62.32 [43]	81.25 [13]	
Trade School/College Degree	35.00 [35]	40.00 [4]	40.00 [2]	37.68 [26]	18.75 [3]	

^a^Analysis of variance and Fisher’s Exact test were used to compare between 4 groups in age and all the other characteristics, respectively.

**Table 3:** Characteristics of participants by providing entire 6 saliva samples without directions violated versus those with directions violated.

	Total Complete Saliva (n = 98)	Complete Saliva without Directions Violated (n = 74)	Complete Saliva with Directions Violated (n = 24)	*p-*value^a^
	M (SD)/% [n]	M (SD)/% [n]	M (SD)/% [n]	
Age	35.74 (15.46)	38.18 (16.21)	28.25 (9.83)	0.0057
Gender				0.3451
Cisgender Male	47.42 [46]	47.95 [35]	45.83 [11]	
Cisgender Female	32.99 [32]	35.62 [26]	25.00 [6]	
Transgender	19.59 [19]	16.44 [12]	29.17 [7]	
Sexual Orientation				0.1899
Monosexual	55.10 [54]	51.35 [38]	66.67 [16]	
Bisexual	44.90 [44]	48.65 [36]	33.33 [8]	
Race/Ethnicity				0.4388
Asian	4.08 [4]	5.41 [4]	0.00 [0]	
Black/African American	35.71 [35]	39.19 [29]	25.00 [6]	
Latino	16.33 [16]	14.86 [11]	20.83 [5]	
Non-Latino White	37.76 [37]	33.78 [25]	50.00 [12]	
Other/Multiracial	6.12 [6]	6.76 [5]	4.17 [1]	
Education				0.6908
≤ High School Diploma	63.27 [62]	62.16 [46]	66.67 [16]	
Trade School/College Degree	36.73 [36]	37.84 [28]	33.33 [8]	

^a^Analysis of variance and Fisher’s Exact test were used to compare between 2 groups in age and all the other characteristics, respectively.

Of 124 participants, 16 participants (12.90%) did not provide any useable GPS data. There were 108 (87.10%) participants who provided at least one day of GPS data and 98 (79.03%) with at least 4 days of data. GPS wear day values varied among the 108 participants with GPS data (M = 7.33; SD = 2.51; Range: 1–16). Fewer than half (n = 50, 40.32%) wore the GPS device one week or less. Slightly over a third of participants wore a GPS device for 8 days (n = 41, 33.06%). Of those with GPS data, there were 17 participants (13.71%) who had GPS data for a period of 9 or more days. Except 2 participants who wore the GPS at least 15 days, participants who wore the GPS 7 days had the highest average daily valid GPS points (i.e., latitude and longitude values) of 453.74 (SD = 1893.28), followed by those who wore the GPS 8 days who had mean of 140.05 (SD = 450.39) valid GPS points per day (Table 4).

**Table 4:** GPS wear time and average daily valid GPS points by different types of data completion.

Number of GPS Wear Days	Overall		Complete GPS Alone (n = 10)		Complete Saliva Alone (n = 10)		Complete GPS and Saliva (n = 88)		Neither GPS nor Saliva (n = 14)	
	% (n)	Mean (SD) of Valid GPS Points/Day	% (n)	Mean (SD) of Valid GPS Points/Day	% (n)	Mean (SD) of Valid GPS Points/Day	% (n)	Mean (SD) of Valid GPS Points/Day	% (n)	Mean (SD) of Valid GPS Points/Day
Missing	12.9 (16)	NA (NA)	0 (0)	NA (NA)	30 (3)	NA (NA)	0 (0)	NA (NA)	81.25 (13)	NA (NA)
1	1.61 (2)	4 (2.83)	0 (0)	NA (NA)	0 (0)	NA (NA)	0 (0)	NA (NA)	12.5 (2)	4 (2.83)
2	2.42 (3)	31.67 (12.27)	0 (0)	NA (NA)	30 (3)	31.67 (12.27)	0 (0)	NA (NA)	0 (0)	NA (NA)
3	4.03 (5)	40.33 (62.7)	0 (0)	NA (NA)	40 (4)	48.67 (69.13)	0 (0)	NA (NA)	6.25 (1)	9 (NA)
4	1.61 (2)	13.38 (10.43)	0 (0)	NA (NA)	0 (0)	NA (NA)	2.27 (2)	13.38 (10.43)	0 (0)	NA (NA)
5	5.65 (7)	62.14 (53.03)	10 (1)	120 (NA)	0 (0)	NA (NA)	6.82 (6)	52.5 (50.93)	0 (0)	NA (NA)
6	5.65 (7)	40.6 (35.47)	10 (1)	39 (NA)	0 (0)	NA (NA)	6.82 (6)	40.86 (38.85)	0 (0)	NA (NA)
7	19.35 (24)	453.74 (1893.28)	10 (1)	61.86 (NA)	0 (0)	NA (NA)	26.14 (23)	470.78 (1933.94)	0 (0)	NA (NA)
8	33.06 (41)	140.05 (450.39)	50 (5)	111.38 (70.21)	0 (0)	NA (NA)	40.91 (36)	144.03 (480.77)	0 (0)	NA (NA)
9	4.84 (6)	115.94 (81.84)	0 (0)	NA (NA)	0 (0)	NA (NA)	6.82 (6)	115.94 (81.84)	0 (0)	NA (NA)
10	1.61 (2)	99.65 (102.04)	0 (0)	NA (NA)	0 (0)	NA (NA)	2.27 (2)	99.65 (102.04)	0 (0)	NA (NA)
11	4.03 (5)	38.53 (17.19)	10 (1)	43.91 (NA)	0 (0)	NA (NA)	4.55 (4)	37.18 (19.54)	0 (0)	NA (NA)
14	1.61 (2)	91.64 (26.67)	0 (0)	NA (NA)	0 (0)	NA (NA)	2.27 (2)	91.64 (26.67)	0 (0)	NA (NA)
15	0.81 (1)	230.47 (NA)	10 (1)	230.47 (NA)	0 (0)	NA (NA)	0 (0)	NA (NA)	0 (0)	NA (NA)
16	0.81 (1)	919.25 (NA)	0 (0)	NA (NA)	0 (0)	NA (NA)	1.14 (1)	919.25 (NA)	0 (0)	NA (NA)
Total	100 (124)	186.14 (935.89)	100 (10)	105.21 (71.37)	100 (10)	41.38 (50.22)	100 (88)	213.03 (1035.49)	100 (14)	5.00 (2.65)

*Note*. SD = Standard Deviation. NA = Non-Applicable.

### Grouping 1: Complete GPS alone

Of the 10 participants with complete GPS data alone who provided socio-demographic information, the average age was 39.80 (SD = 8.79), and majority reported cisgender male (50%, n = 5), Black/African American (60%, n = 6), and less than high school diploma (60%, n = 6) (see Table 1).

The average GPS wear day and average daily GPS points (i.e., latitude and longitude values) in the complete GPS alone group was 8.4 (SD = 2.8, Range: 5–15) and 105.21 (SD = 71.37), respectively. Eighty percent of participants in this group wore the GPS device one week or more (see Table 4).

### Grouping 2: Complete saliva alone

Of 10 participants with complete saliva data alone, the average age was 33.10 (SD = 10.49), and majority reported transgender (50%, n = 5), monosexual (60%, n = 6), Non-Latino White (50%, n = 5), and less than high school diploma (60%, n = 6) (see Table 1).

Half of the participants providing complete saliva samples alone had 1 or 2 samples violated directions. One participant (10%) reported eating within 30 minutes prior to one sample collection, 3 (30%) participants did not freeze 1 out of 6 samples immediately, and 1 (10%) participant reported did not freeze 2 samples immediately.

For those providing fewer than 4 days GPS data but complete saliva samples, the average GPS wear day and average daily GPS points (i.e., latitude and longitude values) was 2.57 (SD = 0.53, Range: 2–3) and 41.38 (SD = 50.22), respectively (see Table 4).

### Grouping 3: Complete GPS and saliva

Of 88 participants with complete GPS and saliva data, the average age was 36.05 (SD = 15.95), and majority reported cisgender male (50.57%, n = 44), monosexual (54.55%, n = 48), Black/African American (37.50%, n = 33) or Non-Latino White (36.36%, n = 32), and less than high school diploma (63.64%, n = 56) (see Table 1).

Within this group, there were 19 (21.6%) participants who violated the directions of saliva sample collection: 11 reported they did not freeze saliva samples immediately, 7 reported eating within 30 minutes prior to sample collections, and 1 reported alcohol use.

The average GPS wear day and average daily GPS points in this group was 7.78 (SD = 1.9, Range: 4–16) and 213.03 (SD = 1035.49), respectively, with 84.09% having worn the GPS device 1 week or more (see Table 4).

### Grouping 4: Neither GPS nor saliva

Of 16 participants with neither complete GPS nor saliva data, the average age was 39.75 (SD = 14.02), and majority reported cisgender male (66.67%, n = 10), bisexual (62.50%, n = 10), Black/African American (53.33%, n = 8), and less than high school diploma (81.25%, n = 13) (see Table 1).

In this group, the average GPS wear day and average daily GPS points was 1.67 (SD = 1.15, Range: 1–3) and 5.00 (SD = 2.65), respectively (see Table 4).

## Discussion

To the best of our knowledge, no study has investigated the feasibility of *combined* use of GPS information and saliva collection among sexual minorities. The incidence of health disparities among individuals identifying as sexual minorities has gained increasing scholarly and theoretical attention [34, 35]. Sexual minorities comprise diverse sexual identities, orientations and practices but as a group tend to be exposed to higher degree of stress from social stigma and discrimination than heterosexuals, resulting in a greater potential for adverse health behaviors and health outcomes [48–52]. Results of the current pilot study indicate that research protocols pairing GPS data collection with participant-collected salivary cortisol data are feasible among this population subgroup as evidenced by high percent of GPS valid days (79.03% had greater than 4 days) and low proportion of completed saliva samples involving direction violations (0.17% - 2.89% of completed saliva samples provided by study team, see Materials and Methods section for more details). Sexual minority individuals provided rich contextual data on their activity paths by wearing a GPS device and on their stress physiology patterns by providing the saliva samples. The GPS device provided automatic collection of data that could be easily synchronized with secondary GIS data on the built environment, and with biological markers of stress as assessed using saliva samples collected with minimal burden and disruption of typical daily routines on participants. The combined used of GPS and saliva samples collection would allow for analyses in examining how changes in the social environment may impact individual physiological stress processes. Although we did not also collect EMA data regarding daily or moment-to-moment mood fluctuations in this protocol, future work could extend into this area to further advance understanding of the interplay between stress-related steroid hormones like cortisol, built and social environmental exposures, real-time self-reported mood data to provide a more comprehensive insight into the experience of sexual minority groups grappling with chronic exposure to stress in their day-to-day lives. GPS data could be collected by the smartphone or via smartphone paired with a dedicated GPS device in order to assess data reliability and possibly remedy any gaps in data that may emerge.

The use of GPS devices has been widely accepted in the general population (typically these are embedded in smartphones). However, relatively few studies employing a GPS device have been conducted in community samples targeting sexual minorities. Despite the apparent convenience of the GPS device wearing and saliva sample collection over the course of the day, it is also important to know about the acceptability of these methods of data collection within a research protocol. Prior work suggests that acceptability can be gleaned by testing the study procedure/protocols, as a dimension of feasibility [77, 78]. Given this, our data could possibly suggest that methods were acceptable to study participants as indicated by high compliance with the protocol/procedures; however, future studies might incorporate feedback and thematically-analyzed qualitative pre- and/or post-interviews to further assess attitudes toward the saliva sample collections and GPS device data collection that may affect use. This would help provide insights that address the complexity of feasibility and potential acceptability of simultaneous use of GPS device and saliva collection in sexual minority communities.

We found that the majority of the participants in this study wore the GPS device, a potential indicator of protocol acceptability, with no significant differences on socio-demographic variables by GPS/saliva compliance groupings, which is consistent with previous research [14, 79–81]. While our study showed 79.03% of participants had at least 4 days GPS data, it is challenging to compare our results with those of other studies due to the longer GPS sampling epoch set up in this study. For example, Duncan and colleagues (2016) showed the feasibility and acceptability of using GPS methods among a sample of young men who have sex with men (YMSM) in New York City. They reported having at least one hour of GPS data (GPS device was set to record information in 30-second intervals) for 1 day, and 84% of their sample had at least 1 hour on 7 or more days for those YMSM (n = 75, 92% 22 or 23 years old) with any GPS data. Another study applied a similar GPS protocol, allowing 10-second epochs with a two-week monitoring period [82]. The high monitoring frequency enhanced the overall quality of the GPS data, and the comparatively longer collection period captured more variations of travel behaviors of the participants [82]. Nevertheless, an important conclusion from these studies—including present study—is that this form of data collection is feasible for research participants from diverse sexual minority groups.

In the current study, there was wide variability in the amount of GPS data that were collected per participant. One obvious contributor was the research design that did not require a specified duration of days, which is a potential limitation of the present study. However, other factors may also be relevant here, including satellite signal loss, atmospheric conditions, and interference from terrestrial objects (multipath error) contributing to missing coordinates [83]. Additionally, it is possible that the device was not worn regularly or daily due to competing interests and activities, including that some participants may have regarded the device as noticeable or cumbersome in some situations (e.g., while exercising, in their place of employment, etc.). Finally, it is worth mentioning that all participants who were screened eligible for participation in this study chose to participate and were not deterred by study procedures, including the impact of GPS data collection on their privacy. However, generally speaking, individuals in historically marginalized groups like sexual minority persons, may possess a heightened sense of intrusion into their privacy from the tracking of their locations [84, 85]. Therefore, future studies with different samples may see greater variability in the acceptability of these procedures. Moreover, results may not generalize to studies that prescribe a “minimum wear time” for the GPS monitoring device inasmuch as that may present additional burden to the participant. This is an important limitation as a recent study recommended 14 wear days for activity path studies, though fewer days may be required depending on the way activity space is measured (e.g., kernel density), environmental attributes of interest to the study, and activity-space representation of the data for analyses [85].

This study also successfully employed saliva collection among a diverse group of adult sexual minorities. Compared to other types of biological samples, saliva is relatively easy to gather. Of 124 participants, more than three-quarters of participants successfully collected 6 entire saliva samples. When accounting for the return of full 6 samples without any violations (e.g., eating food within 30 minutes prior to the collection), compliance dropped from 79.03% of the sample to 58.06% of the sample. Nevertheless, prior studies have required <6 daily saliva samples for analysis (e.g., Cook et al. [38]), suggesting that remaining data may still be valid for analysis after eliminating potential violations without necessitating the listwise deletion of the subject’s remaining saliva data output (e.g., cortisol values).

Other studies among subgroups of the sexual minority community have yielded similarly promising results. For example, in a recent saliva and hair collection feasibility study exploring HIV infection and chronic stress among a sample of 10 transwomen in Puerto Rico, participants were all willing to provide a one-time saliva sample with the assistance of the research team [41–44]. Saliva sample self- or researcher-collection has also been used among young sexual minority men [84, 86], and no problems or concerns were documented in obtaining multiple saliva samples over a longer period of time (e.g., 4 saliva samples each day over the course of 5 consecutive days in a study by Cook et al. [38]). However, it is noteworthy that our study excluded pregnant and lactating women as well as individuals taking medications that could not be postponed during the saliva collection protocol. Thus, feasibility data are limited to individuals who were eligible and/or who agreed to these procedures. Overall, we had 18 ineligibilities (164/182 = 90.1%) with 4 eligible individuals not interested in participation after they heard more about the study. Additionally, the accuracy of cortisol values assayed from saliva samples should be used with caution especially for those participants who reported certain behaviors (e.g., tobacco use and drinking) or non-compliance of saliva collection process (e.g., freeze the sample immediately). However, this represented a relatively low proportion of the completed saliva samples provided to the study team by participants (0.17% - 2.89%).

This feasibility study has limitations. First, the sample was relatively small overall—though large for a feasibility study on a historically marginalized group—and from a single city. The relatively small sample and unique participant characteristics contributed to the possibility that findings cannot be generalized other or all sexual minority adult groups residing in other areas. However, as the principal goal of the study was to determine feasibility, we do not make claims to generalizability. Second, it was not possible to disaggregate the participants’ GPS data by the number of hours participants wore the GPS. Third, no qualitative data regarding the acceptability and perception of GPS and saliva collection were collected from the participants after the assessment period. Such data could have allowed us to assess the burden on sexual minority participants of wearing a GPS or collecting saliva samples. Present work examined compliance with procedures relevant to a convenience sample that consented and enrolled in the study; results cannot determine if the protocol was fully acceptable to participants or that they had no concerns about compliance with it. Fourth, the non-compliance of wearing a GPS device throughout the day could skew the findings in terms of constructing the activity space. Finally, the sexual minority community comprises a diversity of gender and sexual identities; our inclusion criteria was not restrictive in this sense and although we report here on “sexual minorities” generally, it is important to recognize that there may be within-group identification differences (not assessed here) that affect the protocol acceptability or feasibility. Despite these limitations, this study provides important preliminary evidence supporting the combined use of GPS devices and self-collection of saliva samples among individuals identifying as sexual minorities.

The study’s strengths include the unique sample, diverse racial/ethnic composition, inclusion of cisgender men and women and transgender individuals, and the distinctive methodology of the combined use of a GPS device and saliva collection. The simultaneous use of GPS monitors and saliva collection to assess sexual minority individuals’ location and stress biomarkers will allow scientists to investigate the complex effects of the built and social environments and stress. Our findings may reflect characteristics of the sample, which reflects a limited range of population and geographic representation. Further research should investigate the extent to which similar findings might be obtained for sexual minorities in other geographical areas.

## Conclusion

This study demonstrated the feasibility of simultaneous use of a GPS device and collection of saliva samples. GPS methods may be used to measure mobility and environmental exposure, while collected saliva samples may be used for examining stress physiology from diurnal patterns of salivary cortisol. Given the health disparities faced by some sexual minority groups, this feasibility study shed light on the potential use of GPS and saliva to enhance the understanding of the mechanisms underlying the complex interaction between environmental contexts and stress among sexual minority adults. Future research that are powered to investigate causal mechanisms underlying stress dysregulation and the social determinants of health in sexual minority communities is warranted.

## Supporting information

S1 File (XLSX)

## Abbreviations

GPS: Global Positioning System
GIS: Geographic Information Systems
YMSM: Young Men who have Sex with Men
